# Artificial Intelligence in Pediatric Liver Transplantation: Opportunities and Challenges of a New Era

**DOI:** 10.3390/children11080996

**Published:** 2024-08-15

**Authors:** Juri Fuchs, Lucas Rabaux-Eygasier, Florent Guerin

**Affiliations:** 1 Department of General, Visceral and Transplantation Surgery, Heidelberg University Hospital, 69120 Heidelberg, Germany; juri.fuchs@med.uni-heidelberg.de; 2 Department of Pediatric Surgery, Université Paris-Saclay, Assistance Publique Hôpitaux de Paris (AP-HP), Bicêtre Hospital, 94270 Le Kremlin Bicêtre, France; lucas.rabaux@aphp.fr

**Keywords:** pediatric liver transplantation, artificial intelligence, pediatric hepatology

## Abstract

Historically, pediatric liver transplantation has achieved significant milestones, yet recent innovations have predominantly occurred in adult liver transplantation due to higher caseloads and ethical barriers in pediatric studies. Emerging methods subsumed under the term artificial intelligence offer the potential to revolutionize data analysis in pediatric liver transplantation by handling complex datasets without the need for interventional studies, making them particularly suitable for pediatric research. This review provides an overview of artificial intelligence applications in pediatric liver transplantation. Despite some promising early results, artificial intelligence is still in its infancy in the field of pediatric liver transplantation, and its clinical implementation faces several challenges. These include the need for high-quality, large-scale data and ensuring the interpretability and transparency of machine and deep learning models. Ethical considerations, such as data privacy and the potential for bias, must also be addressed. Future directions for artificial intelligence in pediatric liver transplantation include improving donor-recipient matching, managing long-term complications, and integrating diverse data sources to enhance predictive accuracy. Moreover, multicenter collaborations and prospective studies are essential for validating artificial intelligence models and ensuring their generalizability. If successfully integrated, artificial intelligence could lead to substantial improvements in patient outcomes, bringing pediatric liver transplantation again to the forefront of innovation in the transplantation community.

## 1. Introduction

The history of liver transplantation is marked by significant milestones in the field of pediatrics. In 1967, Starzl performed the first successful liver transplant in a 19-month-old child [1]. In 1984, Bismuth and colleagues developed the technique of reduced-size grafts for children awaiting size-matched organs [2]. In 1988, Pichlmayer’s Hannover groups reported the first split liver transplantation, in which both a child and an adult received partial grafts from a single donor liver [3]. The first living-donor liver transplantation in 1989 was performed on a boy receiving a left lobe from his mother [4].

In recent years, however, innovations in liver transplantation (LT) have predominantly been developed and tested in adults, such as types of organ perfusion and recovery. The higher caseloads in adult liver transplantation (aLT), along with ethical and administrative barriers to conducting clinical studies in children, may contribute to this trend [5].

The rapidly growing field of artificial intelligence (AI) could open new opportunities for pediatric liver transplantation (pLT) to lead the way and set new milestones once again. AI has the potential to revolutionize data analysis through machine learning (ML) and deep learning (DL), enabling the handling of complex, multidimensional, and collinear datasets to generate new insights. Crucially for pediatrics, these insights can be obtained without the need for interventional studies, thereby overcoming significant research obstacles in pediatric cohorts. Thus, AI methods appear particularly suitable and promising for advancing research and clinical outcomes in pLT.

This narrative review aims to summarize the existing literature on AI in pLT, provide an overview of its current applications, and discuss the potential opportunities, challenges, and limitations of these methods for improving patient outcomes in pLT.

## 2. Definitions of Artificial Intelligence, Machine Learning, and Deep Learning

In this review, AI is understood as an umbrella term for methods in data analysis that simulate human intelligence based on computer systems [6]. ML is a subset of AI and is referred to as the development of algorithms that enable computer systems to “learn” from and make predictions based on data without direct external instructions. DL is defined as a subset of ML that uses neural networks. These networks are computational models inspired by the human brain and consist of interconnected layers of algorithms that process complex and interdependent data. These networks can “learn” to perform tasks by adjusting the weights based on the data they are trained on, enabling them to recognize patterns and make predictions [6].

## 3. AI in Pediatric Liver Transplantation: Overview of the Existing Literature

**Figure 1:**
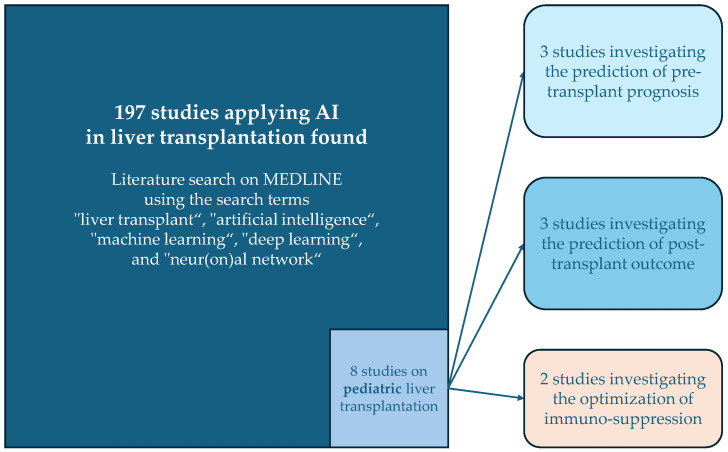
Overview of the results of the literature search and the categories of the studies applying AI in pediatric liver transplantation.

Compared to the volume of studies applying AI in aLT, the field is still in its early stages for pLT. An exploratory literature search on MEDLINE using the search terms “liver transplant”, “artificial intelligence”, “machine learning”, “deep learning”, and “neur(on)al network” identified 197 articles. Only eight of these studies focused on pediatric liver transplantation involving AI (see Figure 1). A summary of these studies is provided in Table 1.

### 3.1. Studies Investigating Pre-Transplant Predictors of Outcome

Given the persistent organ shortage for pediatric recipients [15], risk-adapted prioritization is of utmost importance to improve the prognosis of all children awaiting LT. Currently, a combination of clinical scores (e.g., PELD) and specific diagnoses (e.g., high-urgency listing for hepatoblastoma patients) is used in most transplant regions [16,17]. However, the PELD score has several limitations concerning the listing of patients as well as organ allocation processes. The fact that exception status is granted to a relatively high number of children requiring pLT undermines the PELD scoring system [18]. Moreover, the PELD score has been shown to underestimate the mortality risk of children with high scores, further weakening the reliance on this system [18]. The improvement of listing and allocation systems in pLT seems therefore relevant.

AI can help reveal complex interactions that conventional biostatistical models may overlook. Two studies have applied AI for outcome prediction in children awaiting a liver transplant.

Vodovotz et al. focused on children with acute liver failure (ALF) [7], a unique subgroup where some patients may recover spontaneously without requiring LT, while others risk missing the optimal transplant window. The Pittsburgh group analyzed data from the Pediatric Acute Liver Failure (PALF) study [19], a multi-center, multi-national collaboration. The dataset included 1144 patients under the age of 18 meeting PALF criteria. Systemic inflammatory mediators in serum samples from each of the first seven days after enrollment were analyzed. The authors applied multiple machine learning approaches to identify profiles of spontaneous survivors without LT and age-specific signatures in inflammatory markers. Dynamic Bayesian network inference identified HMGB1 as an important marker across age subgroups. The authors concluded that AI added additional value to their work and that these methods facilitated the identification of inflammatory marker interactions to differentiate between different patient subgroups with PALF. Future steps based on their findings could include the adaptation of treatments according to the identified subgroups. Although the study by Vodovotz et al. has promising implications for future research [7], these findings are not yet sufficient to alter clinical decision-making for PALF patients, highlighting the need for early identification of spontaneous recovery.

Kulkarni et al. applied random forest models to identify predictors of waitlist mortality in children under two years old awaiting LT [8]. They analyzed data from the Scientific Registry of Transplant Recipients (SRTR), a registry of transplant candidates in the United States. Only children < 2 years of age waiting for a first LT between 2002 and 2017 were defined as eligible. They subdivided 4973 patients into three groups based on outcomes: death on the waitlist, spontaneous recovery, and transplant. Baseline variables at the time of listing as well as trajectory variables (changes during waiting time) were analyzed. The authors found that random forest models including trajectory variables had a higher predictive value than those only including baseline variables, with change in serum creatinine emerging as a significant predictor. This is an interesting finding, as renal function is not typically considered critical in pediatric patients compared to adults waiting for LT. This is also mirrored by the fact that renal function is not included in the PELD score [20]. However, the study’s reliance on registry data limits the range of variables and lacks intermediate data between listing and outcome. Moreover, the authors did not compare their ML approach to conventional biostatistical models.

### 3.2. Studies Investigating Post-Transplant Predictors of Outcome

Post-transplant outcomes depend on numerous factors and their interactions. Integrating large amounts and different types of postoperative data, ML can identify risk factors, reveal significant interactions, and find leverage points for targeted treatment, advancing towards individualized therapy for children undergoing pLT. Four studies have applied AI for post-transplant outcome prediction in children. In the following paragraph, they will be discussed in the chronological order of their publication.

Wadhwani et al. used ML to analyze SPLIT registry data from the first year post-transplant to predict ideal outcomes at three years [9]. The authors defined the ideal outcome as being alive at three years with normal ALT (<50 U/L) and GGT (<50 U/L) levels, normal kidney function, no non-liver transplants, no cytopenia, and no PTLD. A total of 334 of 887 (37.7%) included patients reached an ideal outcome at three years. Random forest analyses found non-white race (vs. white race), increased operation time, vascular and biliary complications within 30 days, and duct-to-duct biliary anastomosis as predictors of an unfavorable outcome (no ideal outcome). The authors concluded that AI improved analyses of registry data in their study. Patients at increased risk after pLT were identified, which may help to personalize treatment and improve outcomes in these children. While the study highlights the potential of AI in improving risk stratification, it lacks an evaluation of modifiable risk factors and intervention strategies.

Killian et al. performed a single-center study on children aged 0–18 years undergoing solid organ transplantation (kidney, heart, or liver) between 1988 and 2017 [10]. The sample size for the pLT subgroup was 317. The authors compared ML and DL models to predict 1-, 3-, and 5-year outcomes. Additionally, Shapley additive explanations were applied to increase the interpretability of the DL model results. The DL models did not yield higher predictive values than the ML models in this study, and the interpretability of those models was moderate. The authors concluded that ML and DL are still in their early stages in the field of pediatric organ transplantation. They could be of particular interest for decision-support systems and the identification of high-risk patients needing additional interventions. However, more research is needed to validate initial findings and improve the interpretability and transparency of ML results.

In an ambitious translational work, Ningappa et al. aimed to identify patients at risk of rejection after pLT based on transcriptomics signatures [11]. In a cross-sectional study of 75 children undergoing LT, the authors applied an innovative approach by combining transcriptomics data with protein interactome references to identify network module signatures of patients with and without rejection. State-of-the-art ML methods were used to go one step further and identify which of these modules can potentially be treated by available antirejection drugs. The authors concluded that their AI-based analyses not only identified high-risk patients for rejection after pLT but also found targets for individualized antirejection treatment, which could significantly improve patient outcomes. The study’s retrospective nature implied that the potential targets of individualized immunosuppression had been identified after the occurrence of rejection in the patients. In addition, the heterogenous cohort limits its immediate clinical applicability. Nevertheless, this sophisticated study delivered promising results that lay the foundation for individualized immunosuppressive therapies in pLT.

Prediction of early graft failure was the aim of a single-center study by Jung et al., published in 2022 [12]. In a retrospective analysis of 87 patients undergoing pLT, the authors applied ML in the form of the least absolute shrinkage and selection operator (LASSO) method to identify variables associated with early graft failure. A total of 146 features were included in the models. Four variables were identified as the most predictive features by ML: preoperative hepatic encephalopathy, sodium level at the end of surgery, hepatic artery thrombosis, and total bilirubin level on postoperative day 7. An ML-based logistic regression model with these factors proved to be highly predictive of 90-day graft failure (area under the receiver operating characteristic curve (AUROC) = 0.898 and area under the precision–recall curve (AUPR) = 0.882). The authors concluded that AI helped integrate the high dimensionality and collinearity of the data. A model with high predictive value could be developed that may function as decision support for surgeons and hepatologists, in particular in the early stage after pLT. The retrospective study design and absence of external validation are obvious limitations of this study. However, this work provides valuable insights for future prospective trials to validate these models and eventually test early interventions in high-risk patients. A major strength of the study by Jung et al. is also the comparatively high interpretability of the results compared to other ML-based reports [12].

### 3.3. Studies on Postoperative Immunosuppressive Therapy

Two studies have explored AI’s potential in optimizing immunosuppressive therapy post-pLT. Song et al. investigated tacrolimus concentrations in infants after living-donor LT in a single-center cohort study of 163 patients with biliary atresia and ≤2 years of age [13]. In addition to normal blood values and tacrolimus concentrations, the donors’ and recipients’ CYP3A5 genotypes were tested using PCR. A total of 13 different ML models were applied and compared to predict the tacrolimus concentration 3 months after LT. A ridge regression model yielded the highest predictive value, and the graft-to-recipient weight ratio (GRWR) was the most important variable within that model. The authors concluded that ML improved prediction accuracy in this special patient subgroup and that their results contributed to the individualized dosing of tacrolimus. The limitation of this study is the comparatively low number of included clinical variables, which may decrease the predictive value of the models and lower the added value of ML compared to conventional logistic regression. In addition, the need for genetic testing complicates clinical implementation.

Tan et al. used a small-data AI platform (CURATE.AI) to personalize tacrolimus dosing in a retrospective study of 16 pLT patients [14]. The platform used in this study was built for dose optimization based only on an individual patient’s data and the consequent concentrations to calculate a personalized profile using ML. Doses and tacrolimus concentrations for 16 patients during the first 30 days after pLT were entered. The performance of the AI-driven dosing platform was tested retrospectively by comparing the predicted concentrations to the measured concentrations. Moderate predictive potential was found for the ML-based method (r = 0.45, *p* < 0.001). The authors concluded that the AI-driven platform showed promising potential for providing personalized dosing plans for immunosuppression in the early phase after pLT. The major limitations of the study are the low caseload and the purely retrospective design. Applications in a prospective setting are essential to validating the efficacy of the AI-driven platform in real-world settings.

## 4. Summary of Current AI Applications in Pediatric Liver Transplantation: Research and Clinical Practice

**Figure 2:**
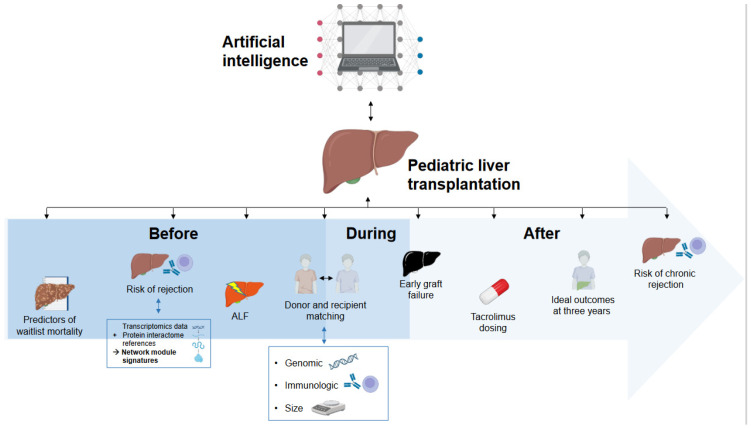
Overview of the current applications of artificial intelligence in pediatric liver transplantation.

The integration of AI in pLT is still in its early stages, but it shows significant promise in enhancing research methodologies and thereby clinical practices. No study was found that reported routine applications of AI in clinical practice in the field of pLT. Current AI applications in research on pLT can be broadly categorized into prediction of outcomes in the pre-transplant phase, post-transplant outcome prediction, and personalized immunosuppressive therapy. Figure 2 provides a graphical overview of the published studies applying AI in pLT (end of the manuscript). Currently, AI has not yet been implemented in clinical practice in pLT, be it in the form of decision support systems or even as independent decision-making tools.

### 4.1. Pre-Transplant Prediction

AI technologies, particularly machine learning (ML) models, have demonstrated potential in predicting outcomes for children on the liver transplant waitlist. The studies by Vodovotz et al. and Kulkarni et al. illustrate how ML can be used to identify critical predictors for specific patient outcomes, such as spontaneous recovery of PALF or waitlist mortality [7,8]. These predictive models may help in better prioritizing patients for transplantation, potentially improving survival rates and optimizing the allocation of scarce organs. AI can uncover complex interactions in clinical data that conventional biostatistics might miss, thereby providing deeper insights into patient conditions and improving decision-making processes. This is of particular relevance for pediatric patients awaiting LT, as there is a far greater number of adult patients on the waiting list who are potentially competing with the children for suitable organs. The options of variant allografts, i.e., split livers and living-donor grafts, have been shown to increase organ availability and decrease waitlist mortality among children awaiting LT [21]. However, these options also increase the complexity of allocation processes, both for donor-recipient matching and organizational aspects [21]. AI may provide valuable tools to overcome this complexity to improve the availability of suitable grafts and optimize the use of donor livers in the future.

### 4.2. Post-Transplant Outcome Prediction

Post-transplant, AI models have been applied to predict overall and specific outcomes, including graft survival, rejection risks, and overall patient health. Studies by Wadhwani et al., Killian et al., Ningappa et al., and Jung et al. have utilized different ML approaches to analyze post-transplant data [9,10,11,12]. These studies have highlighted the potential assets of ML in analyzing complex interactions of different data types that can influence patient outcomes. They offered pathways to more personalized treatments. For instance, the identification of risk factors for early graft failure or rejection through AI may eventually enable clinicians to intervene earlier and more effectively, tailoring post-transplant care to individual patient needs. To date, further studies with more data are needed to validate the initial results and advance the clinical application of AI-based decision-making.

### 4.3. Personalized Immunosuppressive Therapy

The role of AI in optimizing immunosuppressive therapy post-transplant is another promising application, in particular in the vulnerable group of infants undergoing LT, where appropriate dosing is critical to prevent rejection and minimize drug toxicity [22,23]. The studies by Song et al. and Tan et al. have shown that ML models can predict tacrolimus concentrations and help personalize dosing regimens [13,14]. However, the datasets were small, and the study design had a pilot character. AI-driven platforms like CURATE.AI represent a step towards precision medicine, where treatment plans are continuously adjusted based on real-time data from individual patients. These advancements could help health care professionals improve graft functions and improve overall health outcomes for pediatric liver transplant recipients.

## 5. Future Directions and Potential Applications of AI in Pediatric Liver Transplantation

The future of AI in pediatric liver transplantation holds potential for application in other aspects of transplantation that have not yet been explored in children. Several key areas are poised for further development.

### 5.1. Improving Donor-Recipient Matching and Organ Allocation

In adult liver transplantation, AI has been utilized to refine organ allocation processes, in particular donor-recipient matching [6,24,25,26]. The traditional model for organ allocation in adults, the Model for End-Stage Liver Disease (MELD) score [27], has been instrumental in prioritizing patients based on the severity of their liver disease. Despite its widespread adoption, the MELD score has limitations, such as not accounting for certain clinical complications and multi-organ failure, which are significant predictors of mortality [26]. Machine learning (ML) techniques have shown promise in addressing these limitations by integrating a wider array of clinical and laboratory variables [26].

By including not only the recipient data but also the donor data, AI can potentially help optimize donor-recipient matching and improve the accuracy of predictions concerning rejection, complications, and mortality. Translating these advancements into pLT could significantly improve outcomes for children on the transplant waiting list. In addition to organ quality and immunologic aspects, graft size is a vital factor for the outcome of pLT. Thus, the integration of AI-based models including donor, recipient, and imaging data could help create more tailored and accurate risk assessments [28].

### 5.2. Managing Long-Term Complications

AI could also be instrumental in managing long-term complications following pLT. Predictive models could be developed to identify patients at risk of developing chronic rejection, graft dysfunction, or other long-term issues. By analyzing patterns in follow-up data, AI could help tailor follow-up schedules and intervention strategies to individual patient needs, potentially improving long-term outcomes and the quality of life for children.

### 5.3. Enhanced Data Integration

Future AI-based research on pLT could focus on integrating more diverse data sources, including genomic, proteomic, and metabolomic data, to enhance the predictive accuracy of AI models [26]. Combining these data types with clinical and imaging data can provide a more comprehensive understanding of patient health and disease progression. Improved data integration can lead to more robust and accurate predictive models, ultimately improving patient outcomes.

## 6. Limitations and Critical Aspects of AI in Pediatric Liver Transplantation

### 6.1. Trust and Interpretability

One significant challenge in implementing AI is the “black box” nature of ML models, which makes them difficult to interpret [29]. This lack of transparency can reduce trust among clinicians and patients [29,30]. Recent efforts have introduced techniques like Shapley values to enhance model interpretability [30]. However, transparency remains a critical issue in the field of ML-based clinical research. The establishment of regulatory frameworks for AI software, along with efforts to increase explainability and transparency, may increase trust and ensure safety in clinical applications. Such regulations may be conceptualized and introduced by international expert societies in order to guarantee the harmonization of rules and regulations across countries. Certainly, this will also require the support of governments to adopt common legal foundations for data sharing, privacy, and applications of AI in the medical context.

### 6.2. Bias and Data Quality

AI models are only as reliable as the data on which they are trained [31]. If the training data contain inherent biases, these biases can impair the model’s predictions [31,32]. This is particularly concerning in pLT, where caseloads are lower, patient characteristics are more diverse, and factors associated with caregivers additionally influence outcomes. Ensuring model explainability helps researchers identify and correct biases, but ongoing vigilance and method refinement are needed.

In this context, the lack of large-scale, high-quality prospective data should be addressed. While existing databases like the Scientific Registry of Transplant Recipients and the European Liver Transplant Registry provide extensive retrospective data, they often lack detailed clinical information crucial for robust ML model training. To address these potential shortcomings of registry datasets, efforts on several levels are needed. This includes more extensive data collection to provide granular data, rigorous standardization of data collection, and regular training for those involved in data entry. To provide the resources needed to achieve these aims, centralization of data management may help improve the utilization of available funding.

### 6.3. Ethical Considerations and Patient Safety

As AI becomes more integrated into pLT, ethical considerations and patient safety must be considered. Ensuring data privacy and, at the same time, maintaining transparency in AI decision-making processes are critical [33]. Therefore, the cooperation of researchers and clinicians is mandatory to develop guidelines and best practices for the ethical use of AI in pLT. This may include rules for data sharing and protection, clear guidelines concerning the use of AI-based decision support systems, and measured, stepwise implementation of these innovations in clinical practice. Professional societies play a crucial role in this process, as they have both the expertise and the authority among researchers and clinicians to introduce such guidelines. Governments are then called upon to ensure the legal foundations for the international exchange of data and AI applications across borders.

### 6.4. Clinical Implementation

For AI models to truly impact pLT, they must be integrated and validated in clinical settings. Few AI models have been deployed in clinical practice, and those that have typically come from fields like radiology (e.g., AI-supported volumetry) and pathology [28,34]. Integrating AI into clinical workflows requires careful planning, including understandable results, user-friendly interfaces, and control of data quality [6,35]. Prospective evaluations are essential to confirm the efficacy of these tools in real-life settings. These trials will help in assessing the applicability and effectiveness of AI-driven interventions to ensure that these models are generalizable across different patient populations and clinical settings. Finally, there is a need for training and education programs to equip healthcare professionals with the skills to utilize AI effectively [36]. Clinicians, transplant coordinators, and researchers should be trained in the fundamentals of AI, including data management, model interpretation, and ethical considerations.

## 7. Conclusions

In conclusion, the application of AI in pediatric liver transplantation is still emerging, but it holds promise for improving patient outcomes and advancing the field. If current limitations and barriers are addressed, AI can become a valuable tool in the care of pediatric liver transplant patients.

The primary barriers include the need for large, high-quality datasets, the integration of AI systems into existing clinical workflows, and ensuring the interpretability and transparency of AI models. However, as more research validates the benefits of AI in pLT, its adoption is likely to increase, potentially transforming the landscape of pediatric liver transplantation.

By leveraging AI to predict waitlist mortality, enhance donor-recipient matching, recognize early clinical deterioration, personalize post-transplant care, and manage long-term complications, the field of pLT could see substantial improvements in patient outcomes. Future research and clinical trials are needed to validate these approaches and gradually integrate them into routine clinical practice.

## Figures and Tables

**Table 1:** Overview of studies applying artificial intelligence in pediatric liver transplantation.

Authors	Title	Type of Study and Topic	AI Methods	Results/Conclusion	Area
Vodovotz et al., 2020 [7]	Dynamics of Systemic Inflammation as a Function of Developmental Stage in Pediatric Acute Liver Failure	Retrospective multi-center study. Prognostic clinical characteristics and PALF-associated systemic inflammatory mediators in daily serum samples on the outcome of children with acute liver failure.	Dynamic Bayesian network inference, dynamic network analysis	HMGB1 was the sole central node in both INF and NS, aiding in the prediction of pre-transplant prognosis.	Prediction of pre-transplant prognosis
Kulkarni et al., 2021 [8]	Random forest analysis identifies change in serum creatinine and listing status as the most predictive variables of an outcome for young children on liver transplant waitlist	Registry retrospective analysis. Demographic, clinical, listing history, and laboratory variables at the time of listing (baseline variables) and changes in variables between listing and prior to outcome (trajectory variables) were analyzed to predict the outcome of children listed for LT.	Random forest analysis	Change in creatinine, listing status, need for RRT, time spent on the LT waitlist, and type of diagnosis were the most predictive variables.	Prediction of pre-transplant prognosis
Wadhwani et al., 2019 [9]	Predicting ideal outcome after pediatric liver transplantation: An exploratory study using machine learning analyses to leverage Studies of Pediatric Liver Transplantation Data	Registry analysis. Prognostic value of baseline demographic factors and clinical/biochemical factors in the first year post-transplant for prediction of the ideal outcome at 3 years after pLT.	Random forest analyses using ensembles of conditional inference trees	Factors associated with an ideal outcome: white race, shorter duration of operation, absence of vascular and biliary complications within 30 days, absence of pretransplant supplemental feedings, and use of Roux limb biliary anastomosis.	Prediction of post-transplant outcome
Killian et al., 2021 [10]	Machine learning-based prediction of health outcomes in pediatric organ transplantation recipients	Predict 1-, 3-, and 5-year post-transplant hospitalizations using patient and administrative data from a large pediatric organ transplant center.	Naive Bayes, support vector ML, and DL	DL models did not yield superior performance compared to models using ML methods.	Prediction of post-transplant outcome
Ningappa et al., 2022 [11]	A network-based approach to identify expression modules underlying rejection in pediatric liver transplantation	Cohort study. Identification and validation of separate pre- and post-LT transcriptomic signatures of rejection.	Integrative ML approach, combining transcriptomics data with the reference high-quality human protein interactome to identify network module signatures	ML identified high-risk patients for rejection after pLT and also found targets for individualized antirejection treatment.	Prediction of post-transplant outcome
Jung et al., 2022 [12]	Predicting graft failure in pediatric liver transplantation based on early biomarkers using machine learning models	Retrospective single-center cohort study. Identification of predictors of graft failure by ML-based methods.	Least absolute shrinkage and selection operator (LASSO)-based method	The most predictive features were preoperative hepatic encephalopathy, Natrium level at the end of surgery, hepatic artery thrombosis, and POD7 total bilirubin.	Prediction of post-transplant outcome
Song et al., 2022 [13]	Compare the performance of multiple machine learning models in predicting tacrolimus concentration for infant patients with living donor liver transplantation	Retrospective single-center cohort study. Factors influencing tacrolimus concentration in pLT patients.	Thirteen ML algorithms were applied for the development of prediction models. APE, the ratio of APE ≤ 3 ng/mL, and the ideal rate were used to evaluate the predictive performance of the model.	The Ridge regression model (GRWR, donors’ and recipients’ CYP3A5 genotypes, urea, hemoglobin, albumin, and BMI) yielded good predictive performance and provided potential clinical application.	Optimization of immunosuppression
Tan et al., 2022 [14]	Comparing the Performance of Multiple Small-Data Personalized Tacrolimus Dosing Models for Pediatric Liver Transplant: A Retrospective Study	Retrospective single-center study. A small-data, artificial intelligence-derived platform, single-center study using AI to model the dose-response relationship of tacrolimus and identify suitable doses dynamically.	CURATE.AI, a small-data AI-driven platform, uses data from individual patients obtained once daily.	This study established and compared the predictive performance of 6 personalized tacrolimus dosing models for pediatric liver transplant patients and identified a suitable model with consistently good predictive performance based on data from pediatric liver transplant patients.	Optimization of immunosuppression

## Data Availability

The data for this review are publicly available.
